# Parasite-Host Interaction and Pathophysiology Studies of the Human Relapsing Malarias *Plasmodium vivax* and *Plasmodium ovale* Infections in Non-Human Primates

**DOI:** 10.3389/fcimb.2020.614122

**Published:** 2021-02-17

**Authors:** Erica M. Pasini, Clemens H. M. Kocken

**Affiliations:** Department of Parasitology, Biomedical Primate Research Center, Rijswijk, Netherlands

**Keywords:** relapsing malarias, parasite biology, malaria pathophysiology, parasite-host interactions, *Plasmodium vivax*, *Plasmodium ovale*

## Abstract

Malaria remains a serious health concern across the globe. Historically neglected, non-*Falciparum* human malarias were put back on the agenda by a paradigm shift in the fight against malaria from malaria control to malaria eradication. Here, we review the modeling of the relapsing parasites *Plasmodium vivax* (*P. vivax*) and *Plasmodium ovale* (*P. ovale*) in non-human primates with a specific focus on the contribution of these models to our current understanding of the factors that govern parasite-host interactions in *P. vivax* and *P. ovale* parasite biology and pathophysiology.

## Introduction

Malaria is a mosquito-transmitted disease that threatens 3.2 billion people in 95 countries (WHO, 2019 #2). The disease seriously impacts the poorest population living in the tropical and sub-tropical areas of the world. Furthermore, the socio-economic progress of developing countries (Gallup and Sachs, 2001) is hampered by the high mortality combined with an even higher burden of disease, known as morbidity. During the last decade a paradigm shift in the battle against malaria from malaria control to malaria eradication led to renewed attention for the historically neglected non-*Falciparum* malarias, which are known for their tendency to cause morbidity rather than high mortality (Thomson-Luque et al., 2019). Moreover, recent research has shown that the prospects of eradication may be threatened by an increase in the incidence of *non-Falciparum* human malarias, while the overall prevalence of *Plasmodium falciparum*, which is responsible for the high malaria mortality, is declining (Tanner et al., 2015). Here, we review the modeling of the relapsing parasites *Plasmodium vivax* (*P. vivax*) and *Plasmodium ovale* (*P. ovale*) in non-human primates with a specific focus on the contribution of these models to our current understanding of the factors that govern parasite-host interactions in *P. vivax* and *P. ovale* parasite biology and pathophysiology.

### Human *Plasmodium vivax* and *Plasmodium ovale* Malaria

The relapsing human malaria parasites *P. vivax* and *P. ovale* are difficult to distinguish morphologically (Wenk and Stephens, 2000) and have both common and unique characteristics. The natural host of both parasites is man, both parasites are thought to form dormant liver stages [hypnozoites (Garnham et al., 1955; Coatney, 1976; Krotoski, 1985; Soulard et al., 2015)] that unpredictably reactivate in a strain-dependent manner (Battle et al., 2014; Veletzky et al., 2018) to produce relapses (Chin and Contacos, 1966; Chin and Coatney, 1971; Goljan et al., 2003; Centers for Disease and Prevention, 2005; Richter et al., 2010; Morgan et al., 2012; Groger et al., 2017) and form sexual transmissible stages called gametocytes from the very beginning of the sometimes still asymptomatic blood stage infection (Mckenzie et al., 2007; Mueller et al., 2009); both are characterized by a reticulocyte-restricted invasion phenotype (Kitchen, 1938; Rutledge et al., 2017; Thomson-Luque et al., 2019) and a clinical course that had originally been described as mild and benign (Kitchen, 1946; Kitchen, 1949; Collins and Jeffery, 2002; Collins and Jeffery, 2005) but can in fact be very severe (Maguire and Baird, 2010) including spontaneous spleen rupture (Facer and Rouse, 1991; Cinquetti et al., 2010; Zidouh et al., 2017), acute respiratory distress syndrome (Rojo-Marcos et al., 2008; Haydoura et al., 2011; Lacerda et al., 2012; Lau et al., 2013; Guerpillon et al., 2019) and lung injury (Lee and Maguire, 1999; Anstey et al., 2002; Anstey et al., 2007), acute pericarditis (Coton et al., 2011), myocardial infarction (Martin Polo et al., 2020), severe anemia (Erel et al., 1997; Hemmer et al., 2006; Douglas et al., 2012; Johnson et al., 2013; Graciaa et al., 2019), and cerebral malaria (Tanwar et al., 2011).

Due to their reticulocyte restricted phenotypes, in humans both parasites are characterized by relatively low parasitemia levels, rendering their study more complex. However, maximum parasitemia in *P. ovale* is considered to be generally lower than that observed in *P. vivax* malaria. It is widely accepted that both parasites occur globally (Sutherland et al., 2010; Oguike et al., 2011; Rutledge et al., 2017; Battle et al., 2019). However, prevalence differs due to the fact that most *P. vivax* strains are unable to invade Duffy negative red blood cells typical of the African population, while *P. ovale* is independent from the Duffy receptor (Welch et al., 1977). Furthermore, *P. vivax* is geographically the most widely distributed human malaria parasite, characterized by millions of clinical cases every year (WHO, 2016) and responsible for a massive economic burden (Gallup and Sachs, 2001), while large gaps still exist in our knowledge of the incidence and burden of disease for *P. ovale* malaria. Recent advances in sensitive PCR diagnosis show that low-level infections of this parasite seem to be common across malaria-endemic areas, often as complex mixed infections (Sutherland, 2016). However, our understanding of the interactions between *P. ovale* and other malaria parasites, which is essential for the deployment of suitable interventions (Dinko et al., 2013), is poor at best (Mueller et al., 2007; Maguire and Baird, 2010). Most recently two non-recombining sympatric forms of *P. ovale* were distinguished, *P. ovale wallikeri* and *P. ovale curtisi* (Sutherland et al., 2010; Fuehrer and Noedl, 2014). An observational study in British travelers showed that *P. ovale wallikeri* and *P. ovale curtisi* differ significantly in the duration of latency (Nolder et al., 2013). However, the role that the two *P. ovale* species are playing in the clinical presentation of “*P. ovale*” is still not fully defined; thus, hampering the understanding of the role of species differentiation for malaria treatment (Fuehrer and Noedl, 2014). A notable difference between the two forms was the absence of Schüffner’s stippling in *P. ovale wallikeri* blood forms. This finding may guide the differential diagnosis of the two *P. ovale* species (Phuong et al., 2016).

Human *P. ovale* and *P. vivax* infections have been modeled in non-human primates (NHP), either directly or using NHP surrogate parasites, in a number of NHP host species with alternate success as attempts were made to study their pathophysiological characteristics and understand their biology, to investigate co-infections and to establish robust vaccine and drugs testing avenues.

### Modeling of *Plasmodium vivax* and *Plasmodium ovale* Human Malaria Infection in Chimpanzees (*Pan troglodytes*)

The important role of the spleen in parasite-host interactions was confirmed when the first infections with *P. vivax* and *P. ovale* were carried out in intact chimpanzees. While this model supported the development of the liver stages of both parasites, blood stage parasitemia was only transient (Rodhain, 1956; Bray, 1957a; Bray, 1957b; Morris et al., 1996). Upon splenectomy, however, high levels of *P. vivax* blood stage parasitemia were obtained (Young et al., 1975) (Krotoski et al., 1982b) and *P. ovale* parasitemia steadily increased for 10 days with a total duration of patent parasitemia of 21 days (Morris et al., 1996). Notably, the *P. ovale* blood infection in the chimpanzee differed from that in man by its tendency to produce blood schizonts with more than the usual number of merozoites (Collins et al., 1987a). While the role of the aberrant number of merozoites in unclear, it likely is the result of the parasite’s adaptation to its experimental hominidae host. It is unclear whether *P. ovale* related parasite species recently found in great apes (Duval et al., 2009; Mapua et al., 2018) also show this phenotype. Blood stage infections in splenectomized chimpanzees supported mosquito transmission (Young et al., 1975; Morris et al., 1996). However, in this context, parasite strain specific differences were found in the *P. vivax*-chimpanzee model between the Chesson strain and India VII strain. While infections with the former gave rise to variable blood parasitemia and thus, transmission was not always observed (Collins et al., 1986), infections with the latter generated reproducible, high mosquito transmission rates (Sullivan et al., 2001).

Overall, the chimpanzee model was used for two main purposes: 1) modeling host-parasite interactions in a host species closely related to the human host to study the tissue (liver) stages of the *P. vivax* (Bruce-Chwatt et al., 1970; Krotoski et al., 1982b; Nardin et al., 1982; Ferreira et al., 1986; Nardin, 1990) and *P. ovale* parasites (Bray et al., 1963) and 2) obtaining a stable, reproducible and high rate of mosquito transmission to establish a reliable source of parasites for monkey challenge studies and *P. vivax* vaccine development in New World Monkeys (Sullivan et al., 1996). As part of the first objective, numerous *P. vivax* strains, chiefly distinguishable by their biological characteristics, were studied in this model. When it comes to *P. vivax* strains, two main varieties have been defined: the so-called temperate (e.g., North Korean strain) and tropical strains (e.g., Madagascar strain). Differences in exoerythrocytic schizogony were determined in splenectomized chimpanzees through studies of prototypes strains of temperate and tropical strain varieties: compared to the Madagascar strain, which frequently relapsed, the North Korean strain showed a long latency period (Garnham et al., 1975). In the splenectomized chimpanzees, the patterns of these two strains were essentially indistinguishable from those observed in humans (Cogswell et al., 1983). In order to shed light on the different relapse behaviors of these two *P. vivax* parasite strains (Krotoski et al., 1986) a comparison between chimpanzee infections with the *P. vivax* Chesson and North Korean strains through the examination of hepatic biopsies obtained at day 7 and 10 post-infection was carried out. It revealed that in tissue obtained from animals infected with the Chesson strain both pre-erythrocytic schizonts and hypnozoites were present, while only rare hypnozoites at day 7 were observed in animals infected with the North Korean strain. As part of the *P. vivax* studies in chimpanzees it was also found that chimpanzee-passaged *P. vivax* could easily be re-transmitted back to humans (Garnham et al., 1956). The second objective, was considered important as the chimpanzee readily supported the development of infective gametocytes to a much greater extent than did *Aotus* or *Saimiri* monkeys (Collins et al., 1986). In 1990, the group of Mons managed to achieve the mass-scale production of *P. vivax* sporozoites in *Anopheles stephensi* using the chimpanzee as a source of infective blood (Ponnudurai et al., 1990).

Limited blood stage vaccine testing was also carried out in the *P. vivax*-chimpanzee model. The immunization with the merozoite surface protein-1(1–19) (MSP_1–19_) antigens of different *Plasmodium* species, including *P. vivax*, resulted in the detection of antibodies within 2 to 10 weeks post-immunization, which were broadly protective after *P. vivax* challenge in this model (Muerhoff et al., 2010). In another experiment aimed at understanding the efficacy of transmission blocking vaccines based on ookinete surface proteins Pvs25 and Pvs28, blood from chimpanzees infected with the *P. vivax* Salvador (Sal) I strain was mixed with antibodies against the yeast-produced ookinete surface recombinant proteins Pvs25 and Pvs28 and subsequently fed to mosquitoes. As a result, oocyst development in mosquitoes was completely blocked (Tsuboi et al., 2003). However, it is important to consider that chimpanzees are great apes and as such their use in scientific research has been banned, albeit with the exception of extreme scenarios that endanger human health or for the purpose of the preservation of hominidae species, by EU member states (EU, 2010; Altevogt et al., 2012), the UK (Office, 1998), New Zealand (Zealand, 1999), and Australia (Australian Government, 2003). Other states worldwide, such as Japan and the US, have restricted or are in the process of restricting the use of chimpanzees in scientific research (112th Congress, 2011; Kaiser, 2015).

### Modeling of *Plasmodium vivax* and *Plasmodium ovale* Human Malaria Infection in New World Monkeys (*Aotus*, *Saimiri*, and *Challitrix* spp.)

New World monkeys are experimental, but not natural hosts of both *P. vivax* and *P. ovale*, which entails that both parasites need to be adapted to grow in these monkeys. The adaptation of *P. ovale* to grow in *Saimiri* monkeys was partially successful in that mature *P. ovale* liver stages were observed in this host, but no blood stages ensued (Millet et al., 1994b). However, *Aotus* monkeys were found to be refractory to *P. ovale* sporozoite infections (Coatney et al., 1971). Furthermore, both *Aotus* and *Saimiri* monkeys were completely refractory to infection with *P. ovale* infected red blood cells (Coatney et al., 1971; Millet et al., 1994b) from human or chimpanzee origin. This appears to show that host specificity for many malaria parasites occurs at the blood stage level (Millet et al., 1994b). This finding was further supported by studies with the *P. vivax* India VII strain in *Aotus* and *Saimiri* monkeys, which showed that the extended period before parasites appear in the blood stream depends on the difficulty with which exoerythrocytic derived merozoites adapt to heterologous erythrocytes (Sullivan et al., 2001). *Ex vivo* studies using non-human primate red blood cells and a murine anti-Fy6 monoclonal antibody allowed to uncover that the absence of the Fy6 on the Duffy receptor of macaque red blood cells, rendered them resistant to *P. vivax* but susceptible to the NHP malaria *Plasmodium knowlesi*. It was thus the presence of the Fy6 on the Duffy receptor of *Aotus* and *Saimiri* monkeys that made them a good host for *P. vivax* (Nichols et al., 1987; Barnwell et al., 1989). The *P. vivax* proteins binding to the Fy6 on the Duffy receptor were also identified using these models (Wertheimer and Barnwell, 1989). Single-cell gene expression profiles of 9,215 *P. vivax* parasites from bloodstream infections of *Aotus* and *Saimiri* monkeys provided new and robust markers of blood stage parasites, including some that are specific to the elusive *P. vivax* male gametocytes (Sa et al., 2020). While it has been proposed that long-term culture of *P. vivax* in the RBCs of *Saimiri sciureus boliviensis* may provide an alternative to the propagation of *P. vivax* in live animals the use of which is restricted and ethically complicated (Mehlotra et al., 2017), long-term culture of *P. vivax* in these conditions is cumbersome and *Saimiri* monkey RBCs are not widely available. The proteome from two biological replicates of infected *S. s. boliviensis* host reticulocytes undergoing the transition from late trophozoite to early schizont stages showed that a number of host and pathogen proteins contained highly oxidized or nitrated residues, supporting the possibility of oxidative stress in relation to the disease (Anderson et al., 2017). *Ex vivo* experiments using *P. vivax* infected *Saimiri* red blood cells were instrumental in providing evidence for the existence of the parasitophorous duct, establishing a continuity between the environment and the vacuolar space surrounding the intraerythrocytic parasite, which was first considered an *in vitro* artefact. These investigations showed stage and parasite species dependent variations of the characteristics of the duct’s structure (Pouvelle and Gysin, 1997). Using the *P. vivax* Achiote strain, Rossan and Baerg were one of the first to unequivocally demonstrate the presence and development of *P. vivax* exoerythrocytic stages in the liver of *Saimiri* monkeys (Rossan and Baerg, 1975). *P. vivax* model development in *Aotus* and *Saimiri* monkeys aimed at providing parasite-host combinations for chemotherapy, immunology, and biology studies, has provided an opportunity for learning about factors modulating parasite-host interactions and pathophysiology.

Characteristics of the *P. vivax* infections in New World Monkeys do not seem to depend on the donor species (human, chimpanzee or other *Aotus*/*Saimiri* monkey) of sporozoites or blood stage parasites, but appear to be modulated by intrinsic features of the *P. vivax* strain and the immuno-physiology of the recipient host. While the infection of *S. s. boliviensis* with chimpanzee-derived *P. vivax* Chesson strain sporozoites resulted in very long adaptation times (Collins et al., 1987b), the infection with chimpanzee-derived *P. vivax* Sal-I strain resulted in limited adaptation periods, high parasitemia levels, and good reproducibility of infection (Collins et al., 1988). In additional experiments, 67 splenectomized *Aotus azarae boliviensis* were infected with strains of *P. vivax* from Southeast Asia, New Guinea, North Korea, and Central America. Although all strains tested could be adapted to grow in this host, maximum parasitemia levels varied in a strain-dependent manner (VPA, ONG, Chesson, New Guinea, Sal-I > Salvador II > North Korean > Honduras I) and as expected, *P. vivax* strains that were subjected to more blood stage passages partially lost their ability to produce gametocytes and were thus less infective to mosquitoes (Collins et al., 1985c). In a similar experiment, *Aotus vociferans* (karyotype V, Peruvian owl monkey coming from Peru) was also found to be broadly susceptible to a variety of *P. vivax* strains. However, with peak parasite densities ranging from 4,840 to 75,500 per mm^3^, parasitemia levels in this Aotus species were generally lower than in Colombian and Bolivian *Aotus* species (Collins et al., 1987c).

Attempts to adapt a number of different strains show that there is an intrinsic difference in the susceptibility of different *Aotus* subspecies to the same *P. vivax* strain. Sporozoite infections with the *P. vivax* North Korean strain in splenectomized *Aotus lemurinus griseimembra* (Colombia; karyotypes: K-II, K-III, and K-IV) produced higher maximum parasitemia and more readily infected mosquitoes than did infections in *A. a. boliviensis* (Bolivia; karyotype: K-VI) or *A. vociferans* (Peru; K-V) and *Aotus hershkovitzi* (karyotype: K-X) (Collins et al., 1985b). While some level of immunity was expected to develop in the *Aotus*-*P. vivax* North Korean strain combinations, no immunity was observed during primary parasitemia, recrudescence, or relapse in this model (Collins et al., 1985b). Further supporting the notion that the host species plays a key role in modulating parasite adaption, infections with the *P. vivax* Panama strain were more successful in *Aotus nancymaae* (karyotype: K-I) than in other *Aotus* sub-species (Collins et al., 2002), while the *P. vivax* West Pakistan strain could only be established in *A. l. griseimembra*, in which only moderate parasitemia levels were observed (Collins et al., 1981). The *A. vociferans* was completely refractory to infections with sporozoites and only partially susceptible to infections with blood stages of the *P. vivax* ONG/CDC strain isolated from Vietnamese refugees (Collins et al., 1987c), while *A. l. griseimembra* and *A. a. boliviensis* were highly susceptible (Collins et al., 1983). Interestingly, while both *P. vivax* ONG/CDC and NAM/CDC (another strain isolated from Vietnamese refugees) infections in humans readily transmitted to a plethora of mosquitoes, a strongly differential mosquito tropism is observed as a result of *Aotus* infections (Collins et al., 1983). Both Vietnamese refugees’ strains are easily adapted to *A. l. griseimembra* and *A. a. boliviensis* where a long-lasting chronic parasitemia is observed making this host species-parasite combinations potential models for immunological studies into chronic human malaria infections. Splenectomized *A. l. griseimembra* was also suggested to considerably mimicking the course of Chesson malaria in humans in terms of parasitemia and mosquito infection (Collins et al., 2009). Similar differences in susceptibility between subspecies were observed also in *Saimiri* monkeys infected with P. vivax Sal-I where Bolivian phenotype *Saimiri* monkeys (*S. s. boliviensis*) appeared to develop higher peak parasitemia than Peruvian and Guyanan phenotypes (Campbell et al., 1983; Collins et al., 2008). Interestingly, a difference was observed in the *S. s. boliviensis*-*P. vivax* Sal-I model in the virulence of merozoites released by early developing exoerythrocytic schizonts ranging from those producing high-level parasitemia to those inducing low-density avirulent infections (Collins et al., 1996; Arevalo-Herrera and Herrera, 2001). Thus, factors specific to single *S. s. boliviensis* individuals appear to further modulate the virulence of *P. vivax* Sal-I infection in these monkeys. Moreover, a clear difference in susceptibility between Aotus and Saimiri monkeys to the *P. vivax* Sal-I strain was established, which led to questions related to the ability of *P. vivax* Sal-I to invade Saimiri monkeys’ reticulocytes. It was found that while P. vivax Sal-I uses the conventional Duffy binding protein (DBP-I/DBP-II)-mediated pathway to enter *Aotus* monkey red blood cells (McHenry et al., 2010). However, DBP-I is unable to bind to Saimiri monkey red blood cells. Thus, *P. vivax* Sal I must invade Saimiri monkey red blood cells independently of DBP-I/DBP-II. The comparison using RNA sequencing (RNAseq) data of late-stage infections when invasion ligands are expressed in *Aotus* and Saimiri monkey red blood cells, led to the identification of the tryptophan-rich antigen and merozoite surface protein 3 (MSP3) families that were more abundantly expressed in Saimiri monkey compared with *Aotus* monkey infections. These genes may encode potential ligands responsible for *P. vivax* infections of Duffy-negative Africans (Gunalan et al., 2019).

The inoculum size also appears to have an impact on the reproducibility and parasitemia levels in *S. s. boliviensis* infections. While 1,000 sporozoites gave an unreliable rate of infection and 100,000 sporozoites were coupled with an increased number of pathological manifestations due to a very high parasitemia level, the optimum for the Sal-I strain was found to be 10,000 sporozoites (Collins et al., 1988). However, the success in establishing infection also depends on the recipient host’s species/sub-species. A possible role for the skin in modulating the sporozoite infection of *Aotus* and *Saimiri* monkeys becomes apparent when comparing the susceptibility of different *Saimiri* species to mosquito bites and direct i.v. injection of sporozoites. While *S. boliviensis* and *Guyanensis* were found to be uniformly susceptible to both routes of infection, green and black headed *S. peruviensis* were not susceptible to mosquito bites infections, while being susceptible to direct i.v. sporozoite injections (Collins et al., 1994). Although sporozoite infections in *Aotus* and *Saimiri* spp. are known to be poorer producers of *P. vivax* gametocytes than chimpanzee infections (Collins et al., 1986), in New World monkeys the differential ability intrinsic to specific *P. vivax* strains to produce gametocytes appears to be further modulated by the host. While transmission of the *P. vivax* Thai strain could readily be achieved from infected chimpanzees, no transmission was obtained from either *S. s. boliviensis*, *A. nancymaae*, and *A. l. griseimembra* and only limited transmission was obtained after 3 linear passages from *A. a. boliviensis* in multiple attempts using different mosquito species (*Anopheles freeborni*, *Anopheles stephensi*, *Anopheles dirus*, *Anopheles gambiae*) (Collins et al., 1992). While both *Saimiri* and *Aotus* spp. infections with the Thai strain (polymorphic in CSP) resulted in blood stage parasitemia levels similar to other *P. vivax* strains, the reasons for the near-to complete absence of gametocytes from *S. s. boliviensis*, *A. nancymaae*, and *A. l. griseimembra* after limited passages is still a mystery (Collins et al., 1992). This is particularly surprising as in other *Aotus* species a direct relationship between parasitemia counts and mosquito infectivity was observed (Collins et al., 1985b). The Colombian Rio Meta strain, which was adapted to grow in *Aotus* for its unique transmission characteristics (transmissibility to the Central American vector, *Anopheles albimanus*) confirms that a *P. vivax* strain’s ability to produce gametocytes is in part modulated by the host’s subspecies as *A. l. griseimembra* repeatedly showed higher transmission than *A. nancymaae* (Collins et al., 2004). Furthermore, infections in *A. a. boliviensis* with *P. vivax* Sal-II, a strain known to give high mosquito transmission (Collins et al., 1998) from *Aotus* spp., repeatedly and inexplicably failed to infect a number of different mosquito species (Collins et al., 1985a). Importantly, relapses could not reproducibly be established in the New World monkey-*P. vivax* models.

### Studies on the Pathophysiology of *Plasmodium vivax* in *Aotus* and *Saimiri* Monkeys

*Aotus* and *Saimiri* monkeys used in malaria vaccine trials were routinely monitored for parasitemia and general well-being through hematology and clinical chemistry. Detailed histological analyses were carried out at necropsy, which offered a better understanding of the pathophysiology of *P. vivax* malaria in these two NHP species. While a correlation between the lack of protection after malaria challenge and significant changes in hematologic values was found in these studies, the hematological changes observed could not completely be attributed to the increase in the parasite counts or parasite load (Galland et al., 1998). Studies of the organ distribution of developing trophozoite- and schizont-infected red blood cells in such animals showed that the primary site for the infection in both the *Aotus* and *Saimiri* monkey was the splenic vasculature. Secondary organ involvement differed between hosts although some overlap did occur (Fremount and Rossan, 1990).

As a result of in-depth histological analyses of *P. vivax* parasites in organs of infected *Aotus* and *Saimiri* monkeys a considerable portion of immature gametocytes was found in the parenchyma of the bone marrow, while blood stage schizonts were less present in this area of the bone marrow as well as in liver sinusoids. The bone marrow thus appears to be an important reservoir for gametocytes development and proliferation of *P. vivax* parasites (Obaldia et al., 2018).

Of late, the relationship between tissue parasite load and histopathology in a cohort of naive, mostly splenectomized *Saimiri* monkeys (*S. s. boliviensis*) infected *i.v.* with the *P. vivax* Brazil VII strain blood stage parasites was characterized by quantitative parasitological and histopathological analyses. Highest injury levels were found in the lung, liver, and kidney, where pathology was found to be similar to reports from human autopsies. Correlations were found between parasite loads and the intensity of histopathological changes in the lung and liver tissues. In contrast, hemozoin, an inflammatory parasite byproduct was associated with kidney damage, but not directly with parasite load. These findings suggest relevant similarities in the histopathology between *P. vivax* infections in *Saimiri* monkeys and severe *P. vivax* in humans. It was also found that sera from *P. vivax*-infected *Saimiri* monkeys was able to inhibit Fc receptor-mediated phagocytosis and to inhibit ingestion of IgG by normal monkey spleen macrophages. Generally, inhibition was correlated with higher parasitemia (Shear et al., 1987). *In vitro* data obtained using *Saimiri* brain endothelial cells have demonstrated that *P. vivax*-infected red blood cells were able to cytoadhere under static and flow conditions to cells expressing endothelial receptors known to mediate the cytoadhesion of *P. falciparum*. Although *P. vivax*-infected red blood cells’ cytoadhesion levels were found to be 10-fold lower than those observed for *P. falciparum*-infected erythrocytes, the strength of the interaction was similar. Specific antisera directed again *P. vivax* VIR proteins, inhibited the *P. vivax*-infected red blood cells—endothelial cell interaction, thus demonstrating that cytoadhesion of *P. vivax*-infected red blood cells in this model was in part mediated by VIR proteins (Carvalho et al., 2010). Recently, severe thrombocytopenia, which is also observed in human severe *P. vivax* malaria cases, was observed during routine antimalarial drug efficacy trials in *Aotus* monkeys infected with the *P. vivax* AMRU-1 strain. This strain has been considered particularly pathogenic to *Aotus* monkeys, and it was suggested that thrombocytopenia rather than anemia should be regarded as an early indicator of drug treatment failure with this strain (Obaldia, 2007). Although several histological, hematological and clinical chemistry changes in the *P. vivax* infected *Saimiri* and *Aotus* monkeys reflect changes observed in severe *P. vivax* malaria in humans (Peterson et al., 2019), it is important to keep in mind that these models are experimental and thus non-natural models. Moreover, while seminal data are available, more research (e.g., detailed histopathological studies should be performed) would be necessary to validate specific *P. vivax*-new world monkey combinations for use as robust models in the study of the pathophysiology of specific clinical manifestations of human *P. vivax* malaria.

Notably, *Aotus* monkeys have been used in a number of sequential and co-infection studies involving different human malaria parasites. *Aotus* exposed to *P. falciparum* malaria after exposure to *P. vivax* malaria had increased parasitemia and significantly higher levels of mosquito infectivity than naïve monkeys. While monkeys exposed to *P. vivax* malaria after exposure to *P. falciparum* malaria did not show higher parasitemia levels; significantly higher levels of mosquito infectivity were observed also in this case. In *A. azarae boliviensis* infected with *P. vivax*, which had previously been exposed to *P. falciparum* and *Plasmodium malariae* infection higher blood stage malaria was observed with the Vietnam Palo Alto, Chesson and North Korean strains compared to monkeys with no history of malaria (Collins et al., 1985a). Thus, the introduction of another malaria species into a malaria endemic area may result in higher levels of mosquito infection and more rapid establishment and distribution of that species (Collins et al., 1979).

### Surrogate NHP Models of *Plasmodium ovale* and *Plasmodium vivax* Malaria

Over the years *P. vivax* could be adapted to grow in a number of NHP models ranging from the chimpanzee, which is now being phased out of scientific research due to ethical reasons, to the *Aotus* and *Saimiri* monkeys, some of which are endangered species and, as a model in general, marred by limited accessibility due to their ecology. Thus, more accessible models have also been established using surrogate parasites. *Plasmodium cynomolgi* in particular, which is considered the *P. vivax* sister parasite due to their high phylogenetic relatedness (Pasini et al., 2017), has been an excellent and widely used model for *P. vivax*. *Plasmodium cynomolgi* is a simian relapsing parasite, which is readily transmitted by a number of mosquito species (Coatney et al., 1971). Interestingly, while infections of *P. cynomolgi* in humans were considered purely experimental (Eyles et al., 1960), zoonotic occurrences of *P. cynomolgi* malaria transmission to man have recently been reported in South-East Asia (Ta et al., 2014; Imwong et al., 2019). Two natural infection models, the *P. cynomolgi*-*Macaca fascicularis* and the *P. cynomolgi*-*Macaca mulatta* were used in the study of this parasite’s biology and pathophysiology.

*Plasmodium cynomolgi* Berok strain infections with both blood and sporozoite gave rise to extremely low blood stage parasitemia in the *M. fascicularis* model (Collins et al., 1999). However, infections with the *P. cynomolgi* Berok strain in *M. mulatta* induced high-density parasite counts; furthermore, *M. mulatta* readily supported the development of gametocytes infectious to different anopheline mosquitoes routinely maintained in the laboratory (Collins et al., 1999). Of particular interest was the presence of a patterns of every-other-day infectivity of *Anophelines* which persisted often for many days (Coatney et al., 1971). Adding to the versatility of the *Macaca mulatta*-*P. cynomolgi* model, the Berok strain of *P. cynomolgi* can now successfully be maintained in long-term in vitro culture (Chua et al., 2019), where the parasite has been shown to indiscriminately invade monkeys red blood cells (Kosaisavee et al., 2017). Over time different strains were adapted to grow in this model such as the M (Mulligan) and B (*Bastianellii*), the Gombak and the Cambodian strains, and *M. mulatta* (rhesus monkey) became the most used of the NHP species for modeling *P. cynomolgi* infections. Sporozoite induced infections with the B strain were found to peak slightly sooner than those with the M strain. However, the main difference between the two isolates lies in the parasite levels after sporozoite and blood stage inoculation, which are consistently higher for the B strain compared to the M strain indicating that there is an intrinsic biological difference between these two strains (Coatney et al., 1971). Although this model has been used extensively for drug (Flannery et al., 2013; Campo et al., 2015) and vaccine development for *P. vivax* (Galinski and Barnwell, 2008), which is beyond the scope of this review, important studies on hypnozoite biology and pathological features have been performed.

#### Pathophysiology Studies

Experiments with *P. cynomolgi* in *M. mulatta* made it possible to establish the true relapsing nature of this simian parasite (Hawking et al., 1948; Shortt et al., 1948; Shortt and Garnham, 1948a; Shortt and Garnham, 1948b; Shortt and Garnham, 1948c; Shortt et al., 1954). In 1970, Sodeman published the first ultrastructure of the *P. cynomolgi* EE bodies showing no evidence of degenerative changes in the liver cell due to parasitism (Sodeman et al., 1970). Numerous studies were carried out to investigate the origin of relapses, including liver biopsies aimed at confirming the presence of persistent, uninucleate, dormant pre-erythrocytic *P. cynomolgi* stages (Krotoski et al., 1981; Krotoski et al., 1982a; Krotoski et al., 1982c; Bray et al., 1985) or at studying their characteristics (Sinden et al., 1990). As part of these studies, the theory of cyclical development was established (Schmidt, 1986). Infections with the *P. cynomolgi* B strain in this model have been used to study lipid infiltration in host tissues in early (exoerythrocytic) and late (chronic) stages of malaria infection. Histochemically, significant infiltration of neutral and total lipids in liver during the exoerythrocytic stage and in liver and kidney in the erythrocytic stage was demonstrated (Mehrotra and Dutta, 1994). Furthermore, the *P. cynomolgi*-*Macaca mulatta* combination has been instrumental to modeling recrudescence, a hallmark of *P. vivax*, thus once again demonstrating the strong similarity between the two sister parasites (Kocken et al., 2009). Unlike relapse, which is the consequence of the reactivation of dormant liver stages (hypnozoites), recrudescence occurs when parasite blood stages persist at a sub-patent level in drug-treated or untreated individuals followed by a new return to patency. The *P. cynomolgi* model allows for the differentiation between recrudescence and relapse: by treating the *P. cynomolgi* infected *M. mulatta* with drugs selectively targeting *P. cynomolgi* blood stages, such stages can be eliminated, while leaving liver hypnozoites intact. The complete elimination of all blood stages can then be monitored and confirmed by PCR analysis thus enable the differentiation between recrudescence-derived enduring sub-patent blood stage levels and new blood stages derived from the reactivation of liver hypnozoites (Joyner et al., 2015).

While in the past hypnozoite biology of *P. vivax* liver stages could not be studied *in vitro*, thanks to the evolution of a sophisticated *P. cynomolgi ex-vivo* liver stage system using primary hepatocytes of *M. mulatta* (Zeeman et al., 2013), it is now possible to study hypnozoite biology and screen drugs against them *in vitro* and only evaluate the most promising in the *in vivo* model. Most recently, the up-to now lacking proof of hypnozoite reactivation has been obtained in this model using a dual fluorescent *P. cynomolgi* reporter line (Voorberg-van der Wel et al., 2020). *Plasmodium cynomolgi ex-vivo* liver stage cultures in *M. fascicularis* primary hepatocytes have also been used to model hypnozoite reactivation/relapse (Dembele et al., 2014) and for the testing of drugs against the liver stages of relapsing malaria parasites [hypnozoiticidal drugs, (Dembele et al., 2011)].

Clustering analysis on microarrays used to examine gene expression in the *P. cynomolgi* model revealed that a large number of genes involved in RNA processing showed distinct down-regulation during the initial liver phase of infection (Ylostalo et al., 2005). Pathway modeling further showed clear patterns of flux redistribution within the purine pathway of *P. cynomolgi* during infection that are reflected in data from humans infected with *P. falciparum* (Tang et al., 2018). Recent transcriptomic studies provided a novel molecular framework to enhance the understanding of the hypnozoite biology showing that pathways involved in quiescence, energy metabolism, and maintenance of genome integrity remain the prevalent pathways active in mature hypnozoites (Bertschi et al., 2018). Hypnozoites express only 34% of *Plasmodium* physiological pathways, while 91% are expressed in replicating schizonts (Voorberg-van der Wel et al., 2017). The liver-specific protein 2 (LISP2) protein was identified and characterized as an early molecular marker of liver stage development, the expression of which is able to discriminate between dormant hypnozoites and early developing parasites. It was demonstrated that prophylactic drugs selectively kill all LISP2-positive parasites, while LISP2-negative hypnozoites are only sensitive to anti-relapse drug tafenoquine or primaquine (Gupta et al., 2019).

Most recently, an additional model to the *M. mulatta*-*P. cynomolgi* to study hypnozoite-induced relapsing infection has been proposed, namely the Japanese macaque (*Macaca fuscata*)-*P. cynomolgi* B strain (Kawai et al., 2020).

#### Immunological and Physiology Studies

A study in the *P. cynomolgi* model showed that natural killer cell activity profile initially markedly decreased in the earlier stages of infection, but later recovers (Saxena and Biswas, 1990; Sinden et al., 1990). The immune response to *P. cynomolgi* infection in *M. mulatta* seemed to be mediated by anti-parasite, pro-inflammatory responses during primary infection with a transition to protective type 2 responses after repeat infection (Praba-Egge et al., 2002). *Plasmodium cynomolgi* total parasite antigens soluble in culture medium, when injected in *M. mulatta iv*, induced the synthesis and secretion of serum colony-stimulating factors (Singh and Dutta, 1991). *Macaca mulatta* infections initiated by *P. cynomolgi* B strain sporozoites recapitulated pathology of human malaria, including anemia and thrombocytopenia, with inter-individual differences in disease severity (Joyner et al., 2016). Furthermore, *P. cynomolgi* relapse infections can be clinically silent in macaques and thus not cause clinical malaria due to rapid memory B cell responses that help to clear asexual-stage parasites but still carry gametocytes (Joyner et al., 2019). Studies in the *P. cynomolgi* model revealed high levels of reticulocytosis. However, it remains unclear if the parasite itself is able to stimulate this reticulocytosis *via* a parasite-induced factor meant to increase reticulocyte availability and satisfy the parasite’s tropism, or whether it is a physiological reaction of the host to the infection (Fonseca et al., 2018). Concealment of a non-circulating infected RBC sub-population from the peripheral circulation during *P. cynomolgi* infection of *M. mulatta* was also shown, which may be an important aspect of the host-pathogen relationship (Fonseca et al., 2017). Furthermore, using systems analysis to model the alterations in cell populations in the bone marrow during *P. cynomolgi* infection of *M. mulatta* led to the hypothesis that malarial anemia may be driven by monocyte-associated disruption of the GATA-1/GATA-2 in erythroid progenitors resulting in insufficient erythropoiesis during acute infection (Tang et al., 2017).

The *P. cynomolgi* infection model was extensively used to study pathophysiological modifications occurring in pregnancy-related malaria. After *P. cynomolgi* infection, the placenta in *M. mulatta* demonstrated altered physiology. The electron-microscopic observations showed slight complete focal necrosis of the placental tissue, besides alterations in total protein, phosphatases, and proteinases were detected. These changes in cellular constituents of placenta during malaria infection were found to be responsible for the malfunctioning of the placenta and in turn, abnormal fetus development (Saxena et al., 1993). The altered distribution of acid phosphatase and alkaline phosphatase and the resulting changes in hydrolytic enzyme activities in malaria infected early gestational placenta have a direct bearing on functional and morphological integrity of the placental tissue, thus being responsible for early gestational fetal death and abortions (Saxena and Murthy, 2005). Furthermore, alterations able to affect the cellular metabolism, especially steroidogenesis and detoxification process, which in turn would affect the normal development of the fetus as well as maintenance of gestation were found (Saxena and Murthy, 2007).

#### Effects of Interferons, Cytokines, and Steroids on the Biology and Pathophysiology of *Plasmodium cynomolgi*

Prophylactic treatment with recombinant human gamma interferon (rHuIFN 0.1 mg/kg/day) had no protective effect against trophozoite-induced infections, but a strong protective effect against experimental infections with *P. cynomolgi* B sporozoites in *M. mulatta* suggesting that the interferon effect was limited to the exoerythrocytic stage of parasitic development (Maheshwari et al., 1986) thus indicating an inhibition of liver schizogony. Reducing the rHuIFN-gamma treatment in this model to three administrations given close to the time of infection was sufficient to achieve complete protection. However, the protection obtained using rHuIFN-gamma treatment was not enduring and animals were susceptible to reinfection (Puri et al., 1988). IL-1 treatment was effective only when applied before sporozoite inoculation. IL-2 and TNF were effective in higher doses (Maheshwari, 1990). Prophylactic treatment with a single dose of a potent interferon inducer and immune enhancer, 18 h before intravenous inoculation of sporozoites of *P. cynomolgi* B in the *M. mulatta*, completely abolished the infectivity of sporozoites. The inhibitory effect of poly ICLC is dose dependent (Puri et al., 1996). Furthermore, one subcutaneous injection of recombinant human IL-12 2 days before challenge with *P. cynomolgi* sporozoites fully protected *M. mulatta*. In this model rIL-12 protects monkeys through IFN-gamma-dependent elimination of *P. cynomolgi*-infected hepatocytes (Hoffman et al., 1997).

The administration of cortisone in *P. cynomolgi* infected *M. mulatta* monkeys during the primary attack resulted in the marked increase in the peripheral blood infection, while cortisone administration during latent stage or chronic infection caused recrudescences of exceptional severity. Findings suggest that cortisone-induced responses closely compare to reactions to splenectomy. The upsurge in disease derived from cortisone administration appear to result from reduction in the number of macrophages, rather than an inhibition of phagocytic activity as such (Schmidt and Squires, 1951). However, the protracted multiplication of the *P. cynomolgi* M strain in splenectomized *M. mulatta* was often correlated with the appearance of parasites that were less virulent for monkeys with intact spleen than for splenectomized ones. The observed parasite attenuation probably reflects the selection of spontaneously arising mutant characterized by a limited capacity to avoid splenic clearance processes and unscathed or increased capacity to multiply in splenectomized monkeys (Schmidt et al., 1987).

#### Non-Natural Infection Models: *Plasmodium cynomolgi* in New World Monkeys (*Aotus* and *Saimiri* spp.)

Analogously to *P. vivax*, *P. cynomolgi* infection models were successfully established in New World monkeys using the *P. cynomolgi* Berok, Gombak, Smithsonian, Cambodian, and B strains (Collins et al., 1999; Collins et al., 2005b; Sullivan et al., 2006). In general, high density parasite counts, good levels of gametocytes and very efficient transmission were observed. Comparable to *P. vivax* infections in the *Aotus* and *Saimiri* models, studies of different *P. cynomolgi* strains in *Aotus* monkeys show that the characteristics of any specific host-parasite combination depend both on the *P. cynomolgi* strain and on the sub-species of the infected *Aotus* monkey. Furthermore, *Saimiri* monkeys were found to be less susceptible than Aotus in infections with both *P. vivax* and *P. cynomolgi* (Collins et al., 2005b; Sullivan et al., 2006).

### Co-Infection Studies

Co-infections with *P. cynomolgi* and simian immunodeficiency virus (SIV) suggest that such co-infections enhance SIV-induced disease progression and impair the anti-*Plasmodium* immune response (Koehler et al., 2009). SIV infection aggravates malaria pathophysiology in Chinese *M. mulatta*. The animals coinfected with SIV and *P. cynomolgi* had suppressed total CD4^+^ T cells, central memory T cells, and naïve T cells, which may result from the imbalanced immune activation and faster CD4^+^ T cell turnover (Liu et al., 2019).

Co-infection have also been reported between *P. cynomolgi* and *Babesia* spp. In such co-infection there was a significant decrease in *P. cynomolgi* parasitemia, which correlated with increases in the levels of proinflammatory monocytes and with higher C-reactive protein serum levels. Ongoing infection with *Babesia microti* parasites leads to suppression of malaria infection (van Duivenvoorde et al., 2010).

### Neglected NHP-Simian Relapsing Malaria Parasite Models

*Plasmodium* cynomolgi is the most widely used model for relapsing malaria, but some studies have also been performed with other NHP relapsing malarias.

The *Plasmodium fieldi*-*M. mulatta* model has been used historically, but also more recently, for a number of biological studies including as a model for relapsing malaria parasites *P. ovale* and *P. vivax* and the testing of the effects of new radical curative drugs (Martinelli and Culleton, 2018). Experimental infections in *M. mulatta* (Warren et al., 1964) and *M. fascicularis* (Warren and Wharton, 1963) were established. However, the parasite’s blood stage unique staining characteristics are modified in these models. In 1971, relapses in the *P. fieldi*-*M. mulatta* model were first observed and published (Collins and Contacos, 1971). With the exception of *P. cynomolgi*, *P. fieldi* exo-erythrocytic changes in the *M. mulatta* are probably the most extensively studied (Held et al., 1967; Coombs et al., 1968). *Plasmodium fieldi* infection in the *M. mulatta* give rise to the relatively low-level parasitemia in non-spelenectomized *M. mulatta*, which does not appear to endanger the life or well-being of the host (Collins et al., 1984). Higher parasitemias and a marked increase in mosquito infectivity (290) were observed upon splenectomy of *M. mulatta*. The reproducibility of transmission studies using sporozoites of *Anopheles dirus* and *Anopheles Stephensi* mosquitoes suggests that this would be a good model system for a number of studies requiring sporozoite-induced infections (Collins et al., 1973b). In addition, *P. fieldi* antigens display high levels of cross-reactivity to antibodies of other malarias, which makes them useful in sero-epidemiological studies (Salfelder and Mannweiler, 1981; Knobloch et al., 1982; Gebreel et al., 1985).

The name *Plasmodium simiovale* was chosen for this new parasite found in toque macaques (Dissanaike, 1965), its natural host, to underline that it should be considered the simian counterpart of the human malaria parasite *P. ovale*. Experimentally, *M. mulatta* is susceptible to infection either by sporozoite or blood stage *P. simiovale* parasites (Collins and Contacos, 1979); the parasitemia never reaches a high level, even after splenectomy. As shown by the early attempts to cultivate exoerythrocytic stages of *P. simiovale ex-vivo* (Millet et al., 1994a), the *P. simiovale*-*M. mulatta* model, similarly to the P. fieldi-*M. mulatta* model, is considered an interesting model to study the peculiarity of *P. vivax* malaria: the formation of dormant liver stages known as hypnozoites (Collins et al., 1972; Cogswell et al., 1991), which can reactivate at a later time point leading to relapses (Collins and Contacos, 1974). The *P. simiovale* model has also been proposed as a model to study immune evasion mechanisms (Prajapati and Singh, 2014).

*Plasmodium simium* models in New World monkeys are also interesting. *Plasmodium simium* infects both New World monkeys (Coatney et al., 1971) and humans (Brasil et al., 2017). It has been adapted to grow in both intact and splenectomized (higher parasitemia) *Saimiri* and *Aotus* monkeys (Collins et al., 1973a) and has been used as an experimental model of *P. vivax* malaria infections and for the testing of candidate vaccine against malaria (Collins et al., 2005a). Although it remains to be established whether *P. simium* is a genuinely relapsing malaria parasite, its close genetic (Camargos Costa et al., 2015; Lalremruata et al., 2015) and biological (Collins et al., 1969; Collins et al., 1974) relationship to *P. vivax*, which led some authors to refer to it as *P. simium/P. vivax* (Lim et al., 2005; Grigg and Snounou, 2017) suggests that these models may deserve additional studies.

## Discussion

Across time, the availability of NHP models for human malaria parasite infections has driven forward our understanding of parasite biology and pathophysiology through the study of parasite-host interaction and the testing of drugs and vaccines. Different NHP models have emerged as suitable for the study of *P. vivax*, which vary in their accessibility: while the chimpanzee model is being phased out of scientific research due to ethical concern, accessibility to *Aotus* and *Saimiri* models is restricted to specific primate centers due to their ecology and the Convention on International Trade in Endangered Species of Wild Fauna and Flora status of some subspecies. In this context, the *P. cynomolgi*-*M. mulatta* model has emerged as particularly useful for the study of *P. vivax* biology and pathophysiology due to the high phylogenetic relatedness between both the *P. cynomolgi* parasite and *P. vivax* (sister parasite) and its *M. mulatta* host and humans. While this natural infection model has been used extensively for the study of hallmarks of *P. vivax* infections in humans such as recrudescence and relapse, relapses could not be reproducibly established in the *P. vivax*-New World monkey models. However, quantitative parasitological and histopathological analyses carried out in these models, have shown great similarity with the results of human autopsies. Thus, for the study of pathophysiology the *P. cynomolgi* model and the *P. vivax*-*Aotus/Saimiri* models may be complementary.

Overall, it is clear that our knowledge of the hypnozoite biology of the relapsing human malarias has enormously benefitted from the availability of the highly reliable *P. cynomolgi* model. However, while this model well represents *P. vivax* biology and pathophysiology, it is unclear in how far it is suitable for the study of *P. ovale*. As reviewed, a plethora of models are available to study *P. vivax* biology, pathophysiology and for the testing of treatments (vaccines and drugs) against *P. vivax* malaria, but it is clear that the absence of a good and readily available NHP model for *P. ovale* has severely hampered research into this parasite species. In fact, the complete *P. ovale* parasite cycle in the vertebrate host could only be recreated in the chimpanzee, thus severely reducing the avenues available to study the biology and the pathophysiology of this parasite even now that new prospects are created by the recent sequencing of the *P. ovale* genome (Rutledge et al., 2017). In the spirit of finding and validating a suitable NHP model for *P. ovale* and increasing the knowledge of the biology of relapsing malarias in general other NHP models, which have been so far neglected, should be reconsidered and studied more in detail such as the *P. fieldi* and *P. simiovale*-*M. mulatta* and the *P. simium*-Aotus/*Saimiri* models.

## Author Contributions

EP conception and writing of the manuscript. CK discussion, review, and editing of the manuscript. All authors contributed to the article and approved the submitted version.

## Funding

This work was supported by a collaborative grant from the Bill and Melinda Gates Foundation (Grant Numbers OPP1023583 and OPP1141292).

## Conflict of Interest

The authors declare that the research was conducted in the absence of any commercial or financial relationships that could be construed as a potential conflict of interest.
